# Endovascular thrombectomy versus best medical therapy for acute vertebrobasilar artery occlusion in patients with low NIHSS scores: a meta-analysis

**DOI:** 10.1080/07853890.2026.2673631

**Published:** 2026-05-16

**Authors:** Yaqin Qin, Zhuo Min, Guangxin Sun, Hui Xu, Jinghong Lu, Jun Liu

**Affiliations:** ^a^ Department of Neurology, Fuyang People’s Hospital, Fuyang, China; ^b^ Department of Neurology and Centre for Clinical Neuroscience, Daping Hospital, Army Medical Centre of PLA, Army Medical University, Chongqing, China; ^c^ Department of Neurosurgery, Fuyang People’s Hospital, Fuyang, China

**Keywords:** Ischemic stroke, vertebrobasilar artery, National Institutes of Health Stroke Scale, endovascular thrombectomy

## Abstract

**Objective:**

The efficacy of endovascular thrombectomy (EVT) for acute vertebrobasilar artery occlusion (VBAO) presenting with mild symptoms (National Institutes of Health Stroke Scale [NIHSS] score ≤10) remains uncertain. This meta-analysis aimed to compare the effectiveness and safety of EVT versus best medical therapy (BMT) in this population.

**Methods:**

We systematically searched PubMed, Embase, and the Cochrane Central Register of Controlled Trials from inception to September 2025 for comparative studies. The primary outcome was 90-day excellent functional outcome (modified Rankin Scale [mRS] score 0–1). Secondary outcomes included functional independence (mRS 0–2), symptomatic intracranial hemorrhage (sICH), and all-cause mortality. Pooled odds ratios (OR) with 95% confidence intervals (CI) were calculated using a random-effects model.

**Results:**

Seven observational studies involving 3,107 patients were included. In unadjusted analyses, EVT was associated with a higher rate of excellent functional outcome (OR 2.17; 95% CI 1.58–2.96) but not with functional independence (OR 1.58; 95% CI 0.90–2.77). After adjustment for confounders, EVT was associated with higher rate of excellent functional outcome (OR 2.86; 95% CI 1.89–4.31) and functional independence (OR 1.91; 95% CI 1.01–3.62). Safety outcomes including sICH and mortality did not differ significantly between groups.

**Conclusion:**

In patients with acute VBAO and mild symptoms, EVT may be associated with superior functional outcomes compared to BMT alone, without a significant increase in procedural risks. These findings suggest a potential role for EVT in selected patients with low NIHSS scores and underscore the need for confirmation in randomized trials.

## Introduction

Acute vertebrobasilar artery occlusion (VBAO), a critical form of posterior circulation stroke, constitutes approximately 10–20% of all large vessel occlusion-related acute ischemic strokes (LVO-AIS) [1] and is associated with disability and mortality rates exceeding 70% [2]. Recent randomized controlled trials (RCTs), including ATTENTION and BAOCHE, have established endovascular thrombectomy (EVT) as an effective treatment for VBAO patients who have a National Institutes of Health Stroke Scale (NIHSS) score of ≥ 10 [3,4].

However, nearly 30% of VBAO patients present with only mild symptoms (NIHSS ≤10) [5], creating a therapeutic dilemma. Despite mild initial symptoms, these patients remain at considerable risk of early neurological deterioration, which, once irreversible brain tissue injury occurs, is typically associated with poor outcomes. Consequently, delayed or rescue EVT performed after clinical deterioration may fail to confer the same prognostic benefit as early reperfusion. By contrast, EVT is an invasive procedure and carries inherent periprocedural risks, including symptomatic intracranial haemorrhage (sICH) and procedure-related complications [6]. Given that patients with low NIHSS scores generally have a more favourable natural prognosis, the potential harms of EVT may outweigh its benefits and may even result in worse functional outcomes in selected cases. As a result, the optimal treatment strategy for patients with acute VBAO and mild symptoms remains highly controversial [7–13].

This lack of robust, dedicated evidence underscores the urgent need for a systematic evaluation of the benefit-risk profile of EVT in this specific and clinically challenging population. Therefore, this study aims to systematically assess the efficacy and safety of EVT compared with best medical treatment (BMT) in patients with acute VBAO and an NIHSS score ≤10.

## Methods

### Registration and reporting

This review was prepared in line with the Preferred Reporting Items for Systematic Reviews and Meta-Analyses (PRISMA) statement [14], and the study protocol was prospectively registered in the PROSPERO database (CRD420251120138).

### Search and selection criteria

A comprehensive literature search was systematically carried out in PubMed, Embase, and the Cochrane Central Register of Controlled Trials from their inception until September 1, 2025. The aim was to identify studies that compared EVT with BMT among adult patients with VBAO presenting with minor or mild stroke.

Eligible studies were required to satisfy the following conditions: (1) inclusion of adult individuals diagnosed with VBAO and a baseline NIHSS score of 10 or lower; (2) direct comparison between EVT and BMT; (3) reporting of outcomes such as 3-month modified Rankin Scale (mRS) scores and sICH; and (4) use of either RCTs or observational studies. Case reports, as well as animal or *in vitro* investigations, were excluded. Detailed search strategies are provided in Table S1. In addition, reference lists of all eligible articles were manually reviewed to capture further relevant studies that might not have been identified through database searching.

Two reviewers independently extracted data from each included publication. Extracted information encompassed study design features, total sample size, demographic characteristics (age and sex), onset-to-treatment time (OTT), baseline NIHSS score, posterior circulation Alberta Stroke Program Early CT Score (pc-ASPECTS), administration of intravenous thrombolysis (IVT), and all reported efficacy and safety outcomes for both EVT and BMT groups. Any inconsistencies in data extraction were addressed through discussion with the senior author until agreement was reached.

### Risk of bias assessment

For non-RCTs studies, methodological quality was evaluated using the ROBINS-I tool [15], which examines seven potential sources of bias: confounding, participant selection, intervention classification, deviations from intended interventions, missing data, outcome measurement, and selective reporting. All stages of the meta-analysis were conducted independently by two investigators, with disagreements resolved through consultation with a third reviewer.

### Outcome measures

The primary efficacy outcome was excellent functional outcome at 90 days, defined as a mRS score of 0–1 (0 indicated nothing; 6 indicated death). Secondary outcomes included functional independence (mRS 0–2) at 90 days, along with safety outcomes of sICH and all-cause mortality within 90 days.

### Statistical analysis

All statistical analyses were conducted using R software (version 4.3.1). For dichotomous outcomes, pooled estimates were expressed as odds ratios (ORs) with 95% confidence intervals (CIs). Owing to expected clinical and methodological variability across studies, a random-effects model was applied for all meta-analyses. Between-study heterogeneity was evaluated using Cochran’s Q test (*p* < 0.1 considered statistically significant) and quantified with the *I*^2^ statistic, with values exceeding 50% indicating substantial heterogeneity. Sensitivity analyses were performed to validate the robustness of the core findings, including: (1) sensitivity analysis using fully adjusted effect estimates from individual studies. For the adjusted exploratory sensitivity analysis, fully adjusted multivariable ORs and 95% CIs were extracted from the final adjusted models reported in each included study, and pooled using the inverse variance method; (2) sequential leave-one-out sensitivity analyses, in which each included study was removed one at a time to evaluate the influence of individual studies on the pooled effect estimates. Potential publication bias was examined by means of Egger’s regression asymmetry test.

## Results

### Study characteristics and risk of bias

**Figure 1.**
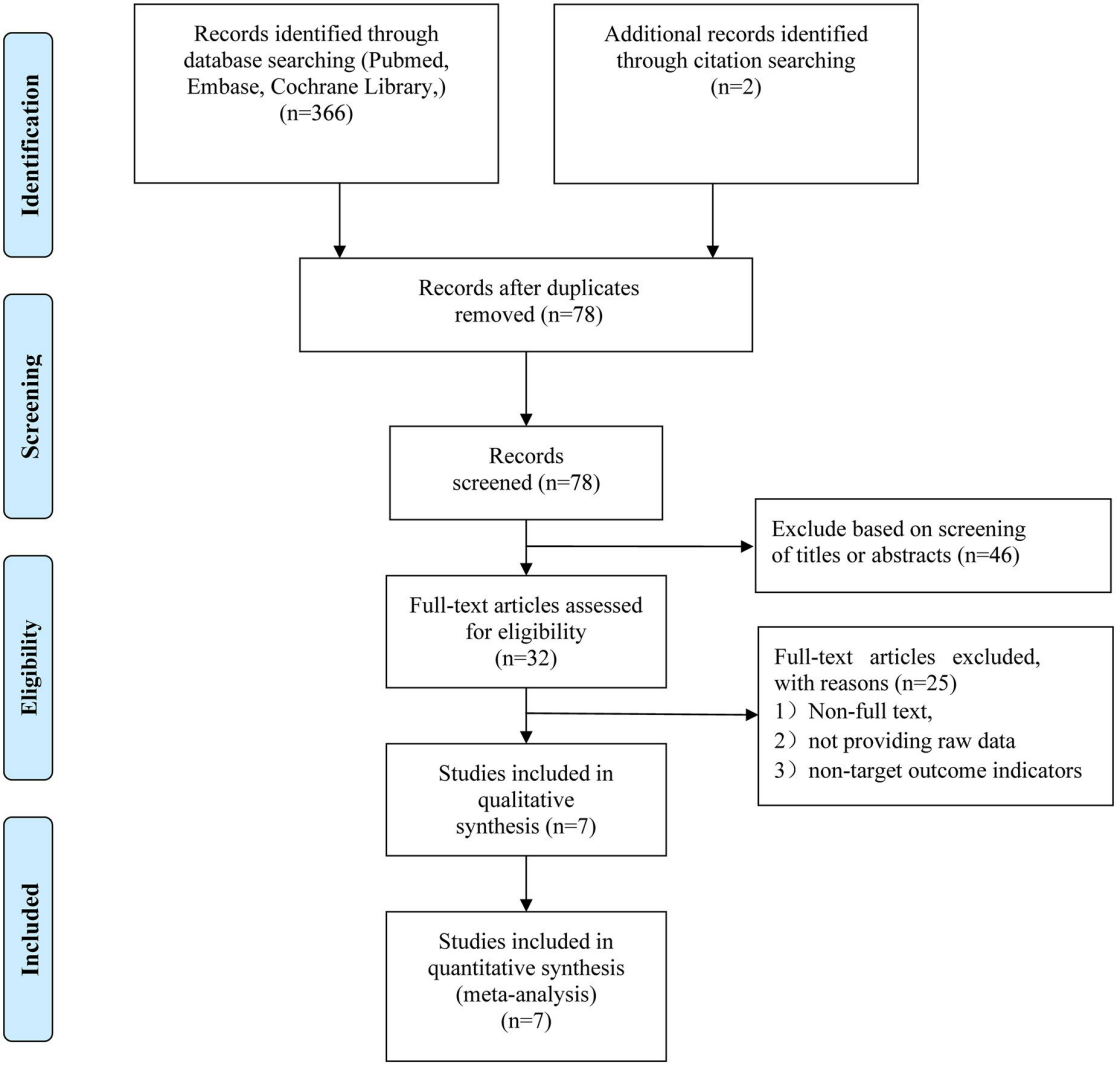
PRISMA flow diagram.

A total of seven cohort studies comprising 3,107 patients who met the inclusion criteria were included in the meta-analysis (Figure 1) [7–13]. Among these, six studies involved patients with basilar artery occlusion (BAO), while one focused on those with VBAO. In three studies, the control group received full IVT, whereas in the remaining four, the control group received BMT. One study restricted inclusion to patients with NIHSS score of 0–5. Across all studies, 1,402 patients received EVT and 1,705 received BMT. The average age ranged from 63 to 71 years in both groups. Table 1 presented the characteristics of the included studies. According to the Risk of Bias tool, all included studies were assessed as having a moderate risk of bias, primarily due to deviations in selection of participants and classification of interventions (Table S2).

**Table 1.** Overview of included studies.

Study	Dargazanli 2024		Kong 2025		Nicolini 2024		Schwarz 2024		Seners 2024		Sun 2024		Xiao 2025	
Country	International		China		International		International		French		China		China	
Design	Prospective study		Retrospective study		Prospective study		Prospective study		Retrospective study		Retrospective study		Retrospective study	
NIHSS score	NIHSS score <10		NIHSS score ≤10		NIHSS score <10		NIHSS score ≤10		NIHSS ≤5		NIHSS score ≤10		NIHSS score <10	
Occlusion site	BAO		BAO		BAO		BAO		BAO		VBAO		BAO	
Imaging methods	MRI or CTA		CTA, MRA or DSA		MRA or CTA		MRA or CTA		NR		CTA, MRA or DSA		CTA, MRA or DSA	
Use of IVT	With or without IVT		With or without IVT		IVT		IVT		IVT		With or without IVT		With or without IVT	
Definition of sICH	ECASS II		HBC		SITS-MOST		SITS-MOST		worsening ≥4 NIHSS points		HBC		HBC	
	EVT	BMT	EVT	BMT	EVT	BMT	EVT	BMT	EVT	BMT	EVT	BMT	EVT	BMT
Participations, n	64	63	70	28	410	354	180	180	28	29	489	897	161	154
Age, years	63.4 ± 16.1	69.0 ± 14.3	64 (55–69)	67 (59–77)	68.3 ± 13.4	67.4 ± 14.4	67.6 ± 13.5	67.9 ± 13.1	67 (56–75)	71 (62–83)	63.9 ± 11.9	66.0 ± 12.4	65 (58–71)	65 (58–73)
Male, n%	39 (60.9)	18 (28.6)	55 (78.6)	21 (75.0)	253 (61.7)	224 (63.3)	64 (35.6)	61 (33.9)	18 (64)	18 (62)	346 (70.8)	605 (69.1)	112 (72.7)	117 (72.7)
pc-ASPECTS	8 (7–9)	8 (7–9)	9 (8–10)	7.5 (6–9)	NR	NR	NR	NR	9 (8–10)	9 (8–10)	9 (8–10)	9 (8–10)	9 (8–10)	9 (8–10)
IVT, n%	24 (37.5)	15 (23.8)	8 (11.4)	5 (17.9)	147 (36.9)	354 (100.0)	142 (78.9)	180 (100.0)	28 (100.0)	29 (100.0)	92 (18.8)	228 (26.0)	28 (17.4)	37 (24.0)
OTT time, minutes	NR	NR	289 (90–410)	122 (82–310)	307 (189–459)	175 (117–279)	175 (131–230)	165 (125–220)	173 (147–243)	205 (180–255)	NR	NR	NR	NR

BAO: basilar artery occlusion; CTA: computed tomography angiography; DSA: digital subtraction angiography; ECASS: European Cooperative Acute Stroke Study; EVT: endovascular thrombectomy; HBC: Heidelberg Bleeding Classification; IVT: intravenous thrombolysis; NIHSS: National Institutes of Health Stroke Scale; NR: not reported; MRA: magnetic resonance angiography; MRI: mgnetic resonance imaging; mRS: modified Rankin Scale; OTT: Onset to treatment; pc-ASPECTS: posterior circulation Acute Stroke Prognosis Early Computed Tomography Score; sICH: symptomatic intracranial hemorrhage; SITS-MOST: Safe Implementation of Thrombolysis in Stroke-Monitoring Study; VBAO: Vertebrobasilar Artery Occlusion.

### Primary outcomes

**Figure 2.**
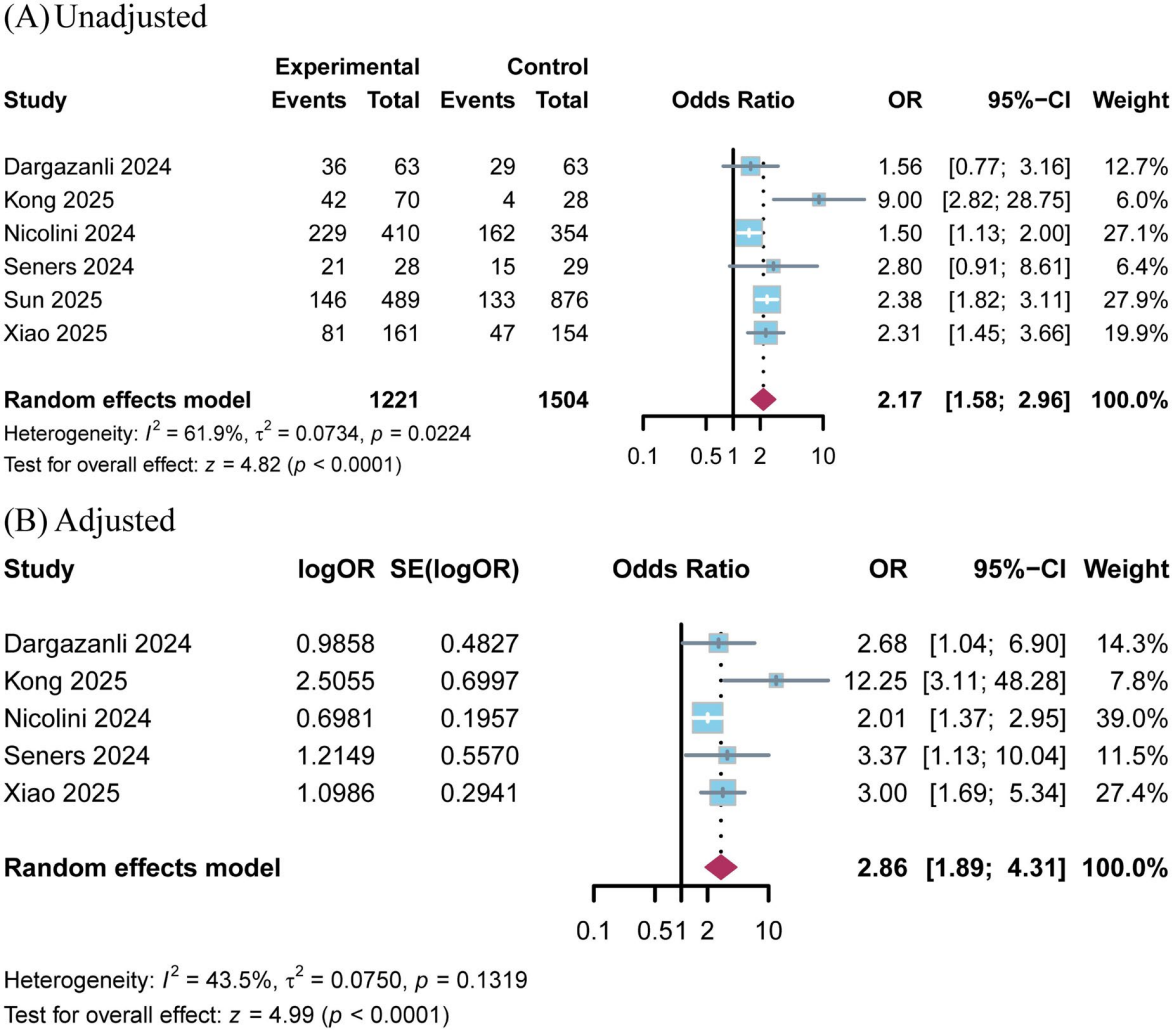
Pooled analysis for excellent functional outcome (mRS 0–1) comparing EVT with BMT groups for VBAO patients with NIHSS ≤10. (A) Unadjusted; (B) Adjusted.

Patients receiving EVT was associated with a higher rate of excellent functional outcome compared to the BMT group (6 studies; OR 2.17; 95% CI 1.58–2.96; *p* < 0.001; *I*^2^ = 62%; Figure 2A).

### Secondary outcomes

**Figure 3.**
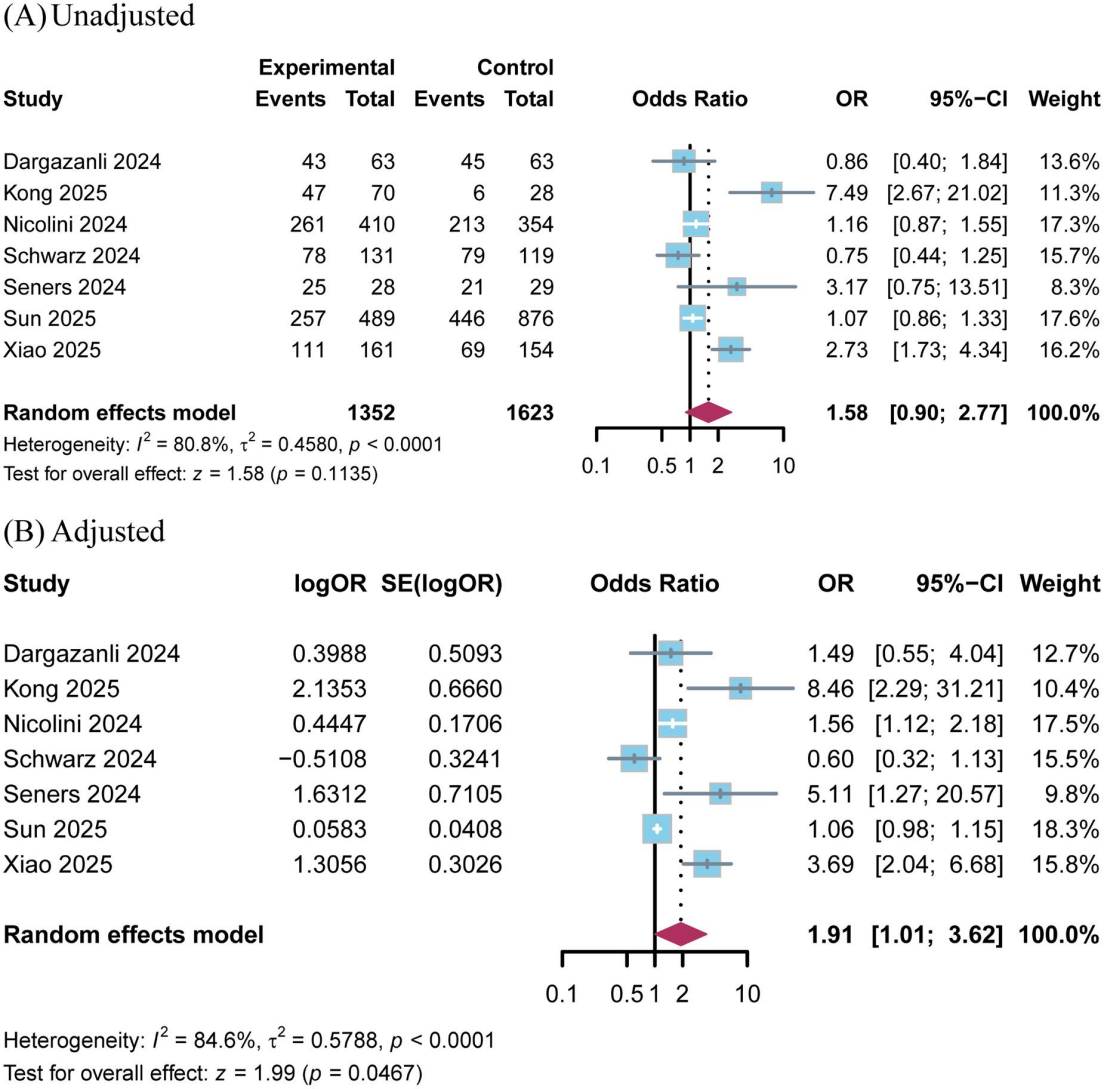
Pooled analysis for functional independence (mRS 0–2) comparing EVT with BMT groups for VBAO patients with NIHSS ≤10. (A) Unadjusted; (B) Adjusted.

When assessing the functional independence at 90 days, meta-analysis of 7 studies revealed no statistically significant difference between EVT and BMT in VBAO patients with NIHSS scores ≤10 (OR 1.58; 95% CI 0.90–2.77; *p* = 0.11; *I*^2^ = 81%; Figure 3A).

No evidence of a difference in the incidence of sICH was observed between the EVT and BMT groups (6 studies; OR 2.00; 95% CI 0.97–4.13; *p* = 0.06; *I*^2^ = 30%; Figure S1A). No evidence of a difference in mortality was observed between the EVT and BMT groups (7 studies; OR 0.89; 95% CI 0.53–1.48; *p* = 0.65; *I*^2^ = 64%; Figure S1B).

### Sensitivity analyses

Sensitivity analysis based on pooled adjusted outcome measures (adjusted for key confounders including age, OTT, and IVT use; detailed in Table S3) demonstrated that compared with the BMT group, patients who received EVT were associated with higher rates of excellent functional outcome (5 studies; OR 2.86; 95% CI 1.89–4.31; *p* < 0.001; *I*^2^ =44%; Table 2 and Figure 2B) and functional independence (7 studies; OR 1.91; 95% CI 1.01–3.62; *p* = 0.047; *I*^2^ =85%; Figure 3B), while no significant differences were observed in sICH (3 studies; OR 1.55; 95% CI 0.44–5.47; *p* = 0.49; *I*^2^ =72%; Figure S1C), or 90-day all-cause mortality (6 studies; OR 0.94; 95% CI 0.71–1.24; *p* = 0.67; *I*^2^ =21%) (Figure S1D).

**Table 2.** The efficacy and safety of EVT compared with BMT.

Outcomes	EVT (*n* = 1404)	BMT (*n* = 1684)	OR (95% CI)	Adjusted OR* (95% CI)
Excellent functional outcome (mRS 0–1)	555/1221	390/1504	2.17 (1.58–2.96)	2.86 (1.89–4.31)
Functional independence (mRS 0–2)	822/1352	879/1623	1.58 (0.90–2.77)	1.91 (1.01–3.62)
sICH	56/1311	32/1604	2.00 (0.97–4.13)	1.55 (0.44–5.47)
90-day all-cause mortality	188/1327	214/1603	0.89 (0.53–1.48)	0.94 (0.71–1.24)

* Outcome after adjusting for confounding factors.

BMT: best medical treatment; CI: confidence interval; EVT: endovascular treatment; mRS: modified Rankin Scale; OR: odds ratio; sICH: symptomatic intracranial hemorrhage.

Leave-one-out sensitivity analyses were conducted for all outcomes (Figure S2). After exclusion of the Nicolini 2024 study, the risk of sICH in the EVT group became statistically significantly increased (Figure S2C). For all other outcomes, the leave-one-out analyses were consistent with the primary results, and the direction of the treatment effect of EVT remained unchanged.

### Publication bias

Egger’s tests did not indicate significant evidence of publication bias for any of the prespecified outcomes (Figure S3).

## Discussion

This meta-analysis synthesized evidence from seven observational studies involving 3,107 patients to evaluate the efficacy and safety of EVT compared with BMT in patients with VBAO presenting with mild symptoms, defined as a NIHSS score ≤10. In unadjusted analyses, EVT was significantly associated with a higher rate of excellent functional outcome, but showed no significant association with functional independence. However, after adjustment for key confounding factors, including age, OTT, and the use of IVT, EVT was associated with both higher rates of functional independence and excellent functional outcome. Notably, no statistically significant differences were observed between the EVT and BMT groups with respect to sICH or 90-day all-cause mortality. Collectively, these findings suggest that mild clinical presentation, as indicated by a low NIHSS score, should not be considered the sole exclusion criterion for EVT in patients with VBAO.

Compared with previous studies, our findings extend the evidence base into an underexplored subgroup. Landmark RCTs such as ATTENTION and BAOCHE focused on patients with NIHSS ≥10 and established the efficacy of EVT in severe VBAO, but their conclusions cannot be generalized to those with milder symptoms [3,4]. Although prior retrospective studies have suggested potential benefits of EVT in patients with low NIHSS scores, their conclusions are often limited by selection bias and ambiguous outcome classification [7–13]. In contrast, based on the findings of this meta-analysis, patients in VBAO who received EVT demonstrated a higher likelihood of achieving excellent functional outcome at 90 days. This suggested that even in patients with minor to mild symptoms, EVT may contribute to neurological improvement through mechanisms such as vascular recanalisation, restoration of cerebral perfusion, and reversal of the ischaemic penumbra [16]. These findings underscored the possibility that patients with acute posterior circulation LVO-AIS, even with low NIHSS scores, may still experience hypoperfusion in functionally critical brain regions. Timely intervention could therefore prevent secondary neurological deterioration.

Notably, the unadjusted analyses showed only a non-significant trend toward improved functional independence with EVT. The apparent strengthening of this association after adjustment suggests that baseline imbalances, such as age, stroke severity, and treatment delays, may partially obscure treatment effects in crude analyses [17]. Nevertheless, given the observational nature of the included studies, these adjusted findings should be interpreted as supportive rather than confirmatory. In addition, although no statistically significant increase in sICH was observed, a directional increase in hemorrhagic risk in certain sensitivity analyses highlights the need for careful patient selection and individualized risk–benefit assessment. These findings underscore the complexity of therapeutic decision-making in mild VBAO, where anatomical vulnerability of the brainstem and heterogeneity in collateral circulation may substantially influence outcomes [18–21].

Current guidelines from the European Stroke Organisation do not recommend EVT over BMT for patients with NIHSS score ≤10 due to a lack of direct supporting evidence [22,23]. Their recommendations are based primarily on subgroup analyses from the RCTs, which included only a limited number of patients with NIHSS score ≤10 and exhibited substantial heterogeneity [24]. In this context, our study provides complementary evidence suggesting that early recanalization may confer neurological benefit even in patients with milder presentations. However, these findings should not be interpreted as definitive evidence of superiority, but rather as hypothesis-generating results that may help inform clinical decision-making. Future randomized controlled trials focusing specifically on mild VBAO populations are warranted, with stratification based on imaging biomarkers (e.g. perfusion status, collateral circulation, and occlusion location) to refine patient selection.

## Limitations

This study has several limitations. First, all included studies were observational in design. Despite multivariable adjustment in our sensitivity analyses, residual and unmeasured confounding remains inherent to non-randomized research, precluding definitive causal inferences. Heterogeneity in covariate selection and adjustment strategies across studies also limits the comparability of these exploratory adjusted effect estimates. Second, the lack of individual patient data restricted our ability to conduct granular subgroup analyses or identify key effect modifiers, including the optimal NIHSS threshold, collateral status, and age-specific treatment effects. Third, notable heterogeneity in sICH definitions across studies may have introduced variability in reported safety events, limiting the interpretability of pooled safety estimates. Fourth, theoretical methodological risks remain, including potential overestimation of effective sample size from using pre-weighting counts in the unadjusted analysis of the inverse probability of treatment weighting based study, and unstable effect estimates from studies with small control groups. Finally, insufficient data prevented a robust head-to-head comparison of EVT versus IVT alone, limiting conclusions on the optimal reperfusion strategy for this population. Taken together, these limitations underscore the need for cautious interpretation of the findings and highlight the importance of future well-designed prospective randomized controlled trials to validate these results.

## Conclusion

This study found that EVT may be associated with improved functional outcomes in patients with mild VBAO stroke compared to BMT. These findings support the potential benefit of EVT in this population, though they require validation in future RCTs.

## Supplementary Material

Supplemental material.docx

## Data Availability

The datasets used and/or analyzed during the current study are available from the corresponding author on reasonable request.
