# Supplementary material for: Endovascular thrombectomy versus best medical therapy for acute vertebrobasilar artery occlusion in patients with low NIHSS scores: a meta-analysis
Source: Ann Med. 2026 May 16;58(1):2673631. doi: 10.1080/07853890.2026.2673631 (PMC13182163; doi:10.1080/07853890.2026.2673631)
Supplement: Supplemental material.docx [file IANN_A_2673631_SM8115.docx]

**Supplemental Material**

| **Contents:** | **Page** |
| --- | --- |
| **Table S1.** Search strategy. | 2 |
| **Table S2.** Risk of bias assessment for non-randomized-controlled trials. | 3 |
| **Figure S1.** Forest plot for safety outcomes. | 4-5 |
| **Table S3.** Confounding factors adjusted in the sensitivity analysis and the definition of symptomatic intracranial haemorrhage. | 6 |
| **Figure S2.** Leave-one-out sensitivity analyses. | 7 |
| **Figure S3.** Egger’s tests for publication bias. | 8-9 |

**Table S1.** Search strategy.

| **P**  Participants | #1.  "stroke"[MeSH Terms] OR "stroke"[Title/Abstract] OR "cerebrovascular"[Title/Abstract] OR "brain vascular"[Title/Abstract] OR "apoplexy"[Title/Abstract] OR "brain ischemia"[Title/Abstract] OR "brain ischaemia"[Title/Abstract] OR "cerebral ischemia"[Title/Abstract] OR "cerebral ischaemia"[Title/Abstract] OR "cerebral infarction"[Title/Abstract] OR "brain infarction"[Title/Abstract] OR "vascular accident"[Title/Abstract] OR "vertebrobasilar artery occlusion"[Title/Abstract] OR "basilar artery occlusion"[Title/Abstract] OR "posterior circulation"[Title/Abstract] |
| --- | --- |
| **I**  Interventional arm | #2.  "thrombectomy"[MeSH Terms] OR "thrombectomy"[Title/Abstract] OR "mechanical clot disruption"[Title/Abstract] OR "mechanical clot disruption"[Title/Abstract] OR "stent retriever"[Title/Abstract] OR "stentriever"[Title/Abstract] OR "endovascular"[Title/Abstract] |
| **C**  Comparison arm | #3.  "thrombolytic therapy"[MeSH Terms] OR "thrombolytic therapy"[Title/Abstract] OR "intravenous thrombolysis"[Title/Abstract] OR "thrombolysis"[Title/Abstract] OR "standard medical treatment"[Title/Abstract] OR "medical management"[Title/Abstract] OR "best medical management"[Title/Abstract] OR "medical treatment"[Title/Abstract] OR "best medical treatment"[Title/Abstract] |
| **O**  Outcome | #4.  Outcome: modified Rankin Scale score or symptomatic intracranial hemorrhage (“mRS”, “modified Rankin Scale”, “sICH”, “intracranial hemorrhage”, “cerebral hemorrhage”, “intracranial haemorrhage”, “cerebral hemorrhage”) |

**Table S2.** Risk of bias assessment for non-randomized-controlled trials.

| **Domain** | **Assessment by outcome** | | | | | | |
| --- | --- | --- | --- | --- | --- | --- | --- |
|  | **Dargazanli 2024** | **Kong 2025** | **Nicolini 2024** | **Schwarz 2024** | **Seners 2024** | **Sun 2024** | **Xiao 2025** |
| Bias due to confounding | Low risk | Low risk | Low risk | Low risk | Low risk | Low risk | Low risk |
| Bias in selection of participants into the study | Moderate risk | Moderate risk | Moderate risk | Moderate risk | Moderate risk | Moderate risk | Moderate risk |
| Bias in classification of interventions | Moderate risk | Moderate risk | Moderate risk | Moderate risk | Moderate risk | Moderate risk | Moderate risk |
| Bias due to deviations from intended interventions | Low risk | Low risk | Low risk | Low risk | Low risk | Low risk | Low risk |
| Bias due to missing data | Low risk | Low risk | Low risk | Low risk | Low risk | Low risk | Low risk |
| Bias in measurement of outcomes | Low risk | Low risk | Low risk | Low risk | Low risk | Low risk | Low risk |
| Bias in selection of the reported result | Low risk | Low risk | Low risk | Low risk | Low risk | Low risk | Low risk |
| Overall | Moderate risk | Moderate risk | Moderate risk | Moderate risk | Moderate risk | Moderate risk | Moderate risk |

**Figure S1.** Forest plot for safety outcomes.

| 1. Unadjusted analysis for symptomatic intracranial hemorrhage. |
| --- |
| 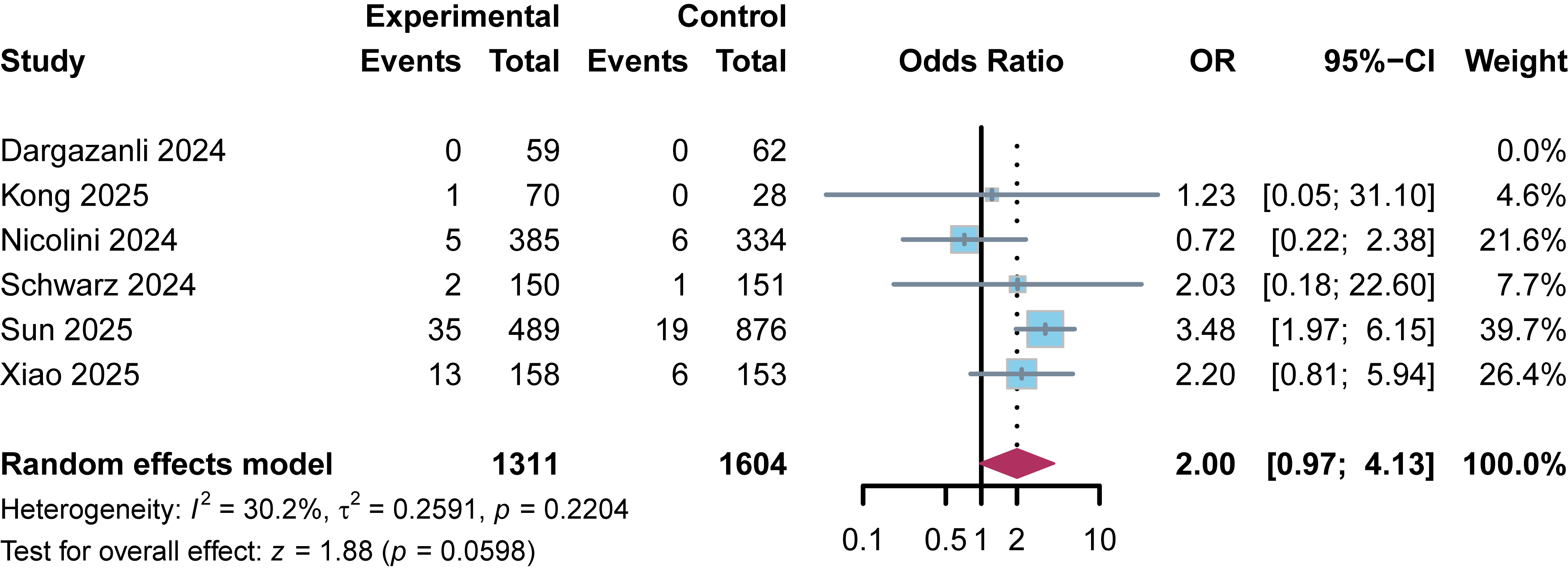 |
| 1. Unadjusted analysis for 90-day all cause mortality. |
| 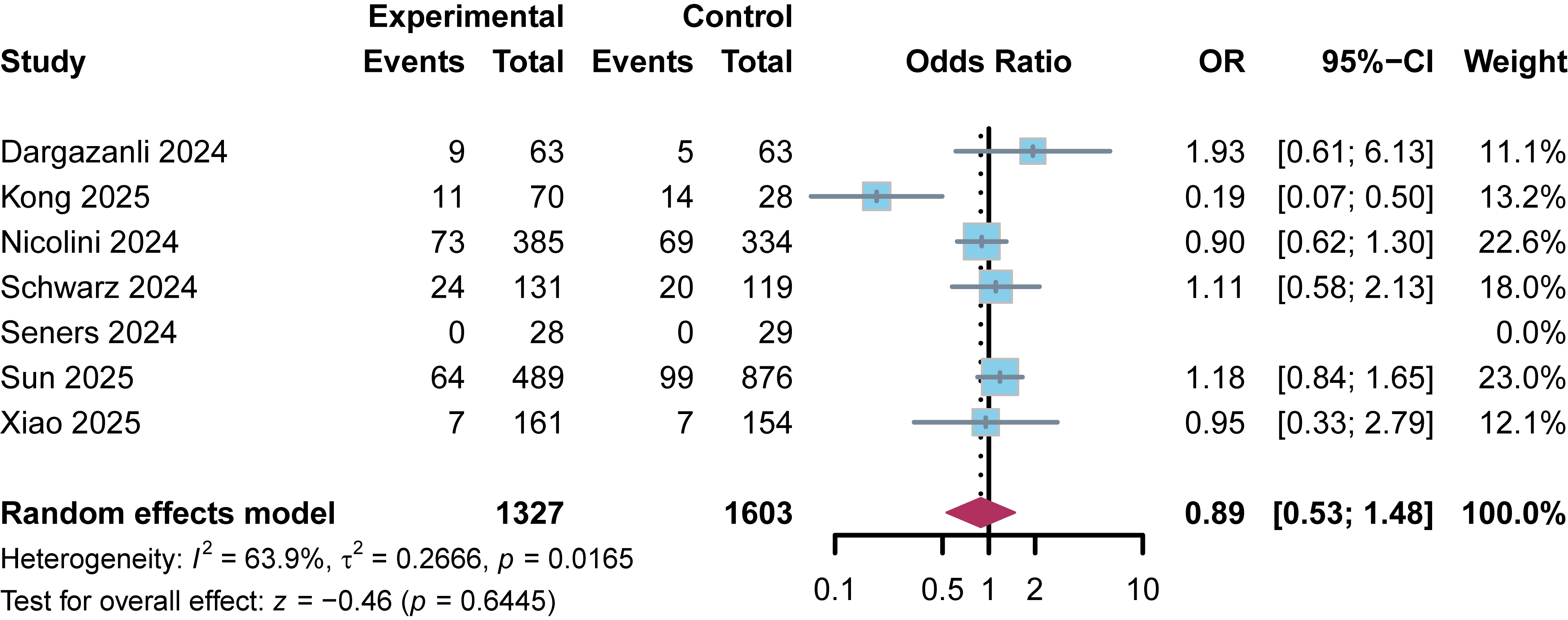 |
| 1. Adjusted analysis for symptomatic intracranial hemorrhage. |
| 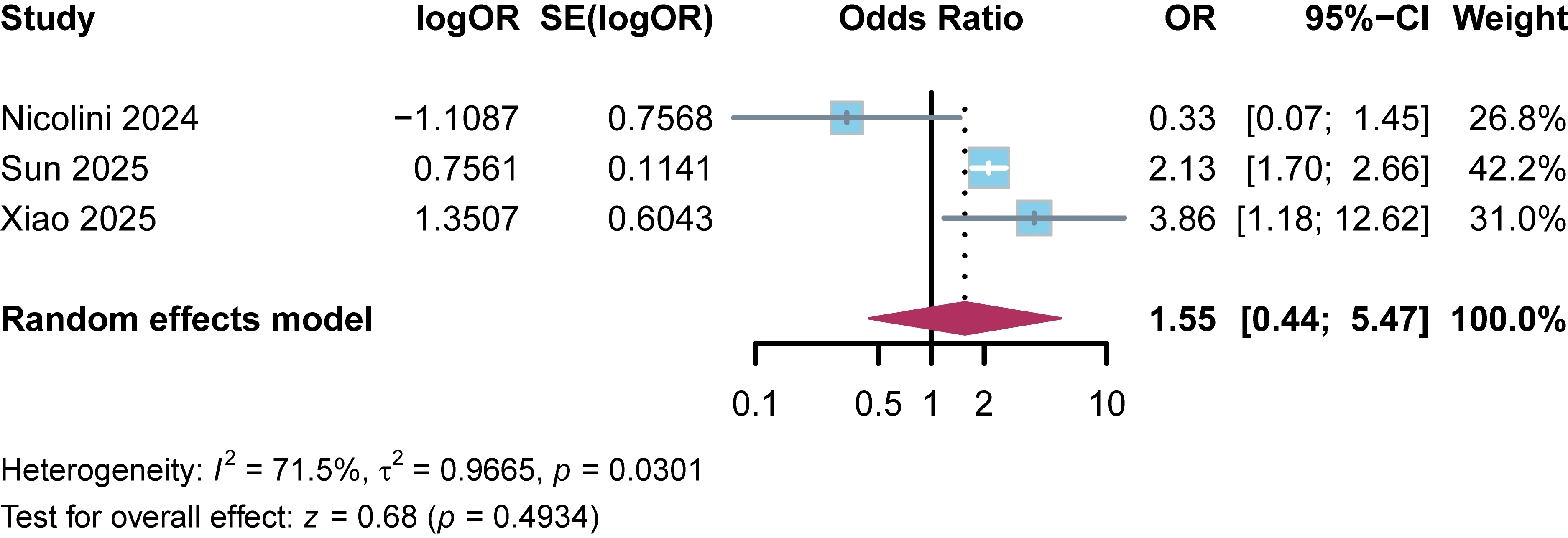 |
| 1. Adjusted analysis for 90-day all cause mortality. |
| 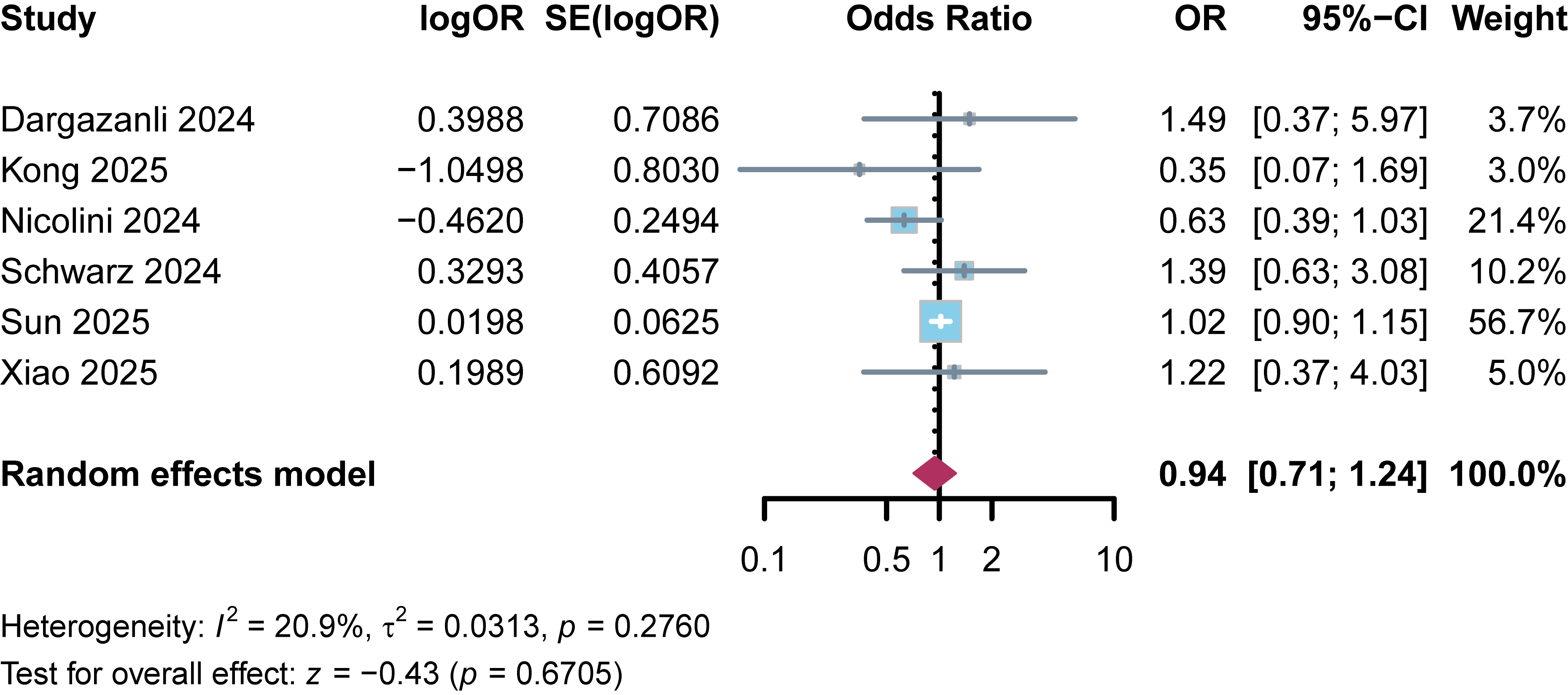 |

**Table S3.** Confounding factors adjusted in the sensitivity analysis and the definition of symptomatic intracranial haemorrhage.

| **Study** | **Confounding factors** | **Definition of sICH** |
| --- | --- | --- |
| Dargazanli 2024 | admission NIHSS score | ECASS II |
| Kong 2025 | age, sex, hypertension, diabetes mellitus, hyperlipidemia, smoking status, previous stroke, coronary artery disease, atrial fibrillation, time from stroke onset to treatment, baseline NIHSS score, stroke etiology, IVT±, and occlusion location | Heidelberg Bleeding Classification |
| Nicolini 2024 | age, sex, onset-to-treatment time, hypertension, diabetes, hypercholesterolemia, history of TIA or stroke in the past 3 months, atrial fibrillation, heart failure, smoking habit, baseline NIHSS score, and site of BAO | ECASS II |
| Schwarz 2024 | age, gender, baseline NIHSS score, pre-stroke mRS, arterial hypertension, hyperlipidemia, diabetes, atrial fibrillation, antithrombotic drug onset to treatment time | SITS-MOST |
| Seners 2024 | age and pc-ASPECTS | worsening ≥4 NIHSS points |
| Sun 2024 | age, sex, hypertension, diabetes, hyperlipidemia, coronary artery disease, previous ischemic stroke/TIA, atrial fibrillation, smoking history, baseline NIHSS score, occlusion site, pc-ASPECTS, intravenous thrombolysis, systolic blood pressure, and time last seen well. | Heidelberg Bleeding Classification |
| Xiao 2025 | age, baseline NIHSS, TOAST classification, number of occlusion sites, atrial fibrillation history, and baseline pc-ASPECTS | Heidelberg Bleeding Classification |

BAO=basilar artery occlusion; ECASS II=European Cooperative Acute Stroke Study II; IVT: intravenous thrombolysis; NIHSS=National Institutes of Health Stroke Scale; mRS=modified Rankin Scale; pc-ASPECTS=posterior circulation Acute Stroke Prognosis Early Computed Tomography Score; SITS-MOST=Safe Implementation of Thrombolysis in Stroke-Monitoring Study; TIA=Transient Ischemic Attack; TOAST=Trial of ORG 10172 in Acute Stroke Treatment.

**Figure S2.** Leave-one-out sensitivity analyses.

| 1. Unadjusted analysis for excellent functional outcome. |
| --- |
| **** |
| 1. Unadjusted analysis for functional independence. |
| **** |
| 1. Unadjusted analysis for symptomatic intracranial hemorrhage. |
|  |
| 1. Unadjusted analysis for 90-day all cause mortality |
| **** |

**Figure S3.** Egger’s tests for publication bias.

| 1. 90-day modified Rankin scale score of 0-2 (*P*=0.2585). |
| --- |
| 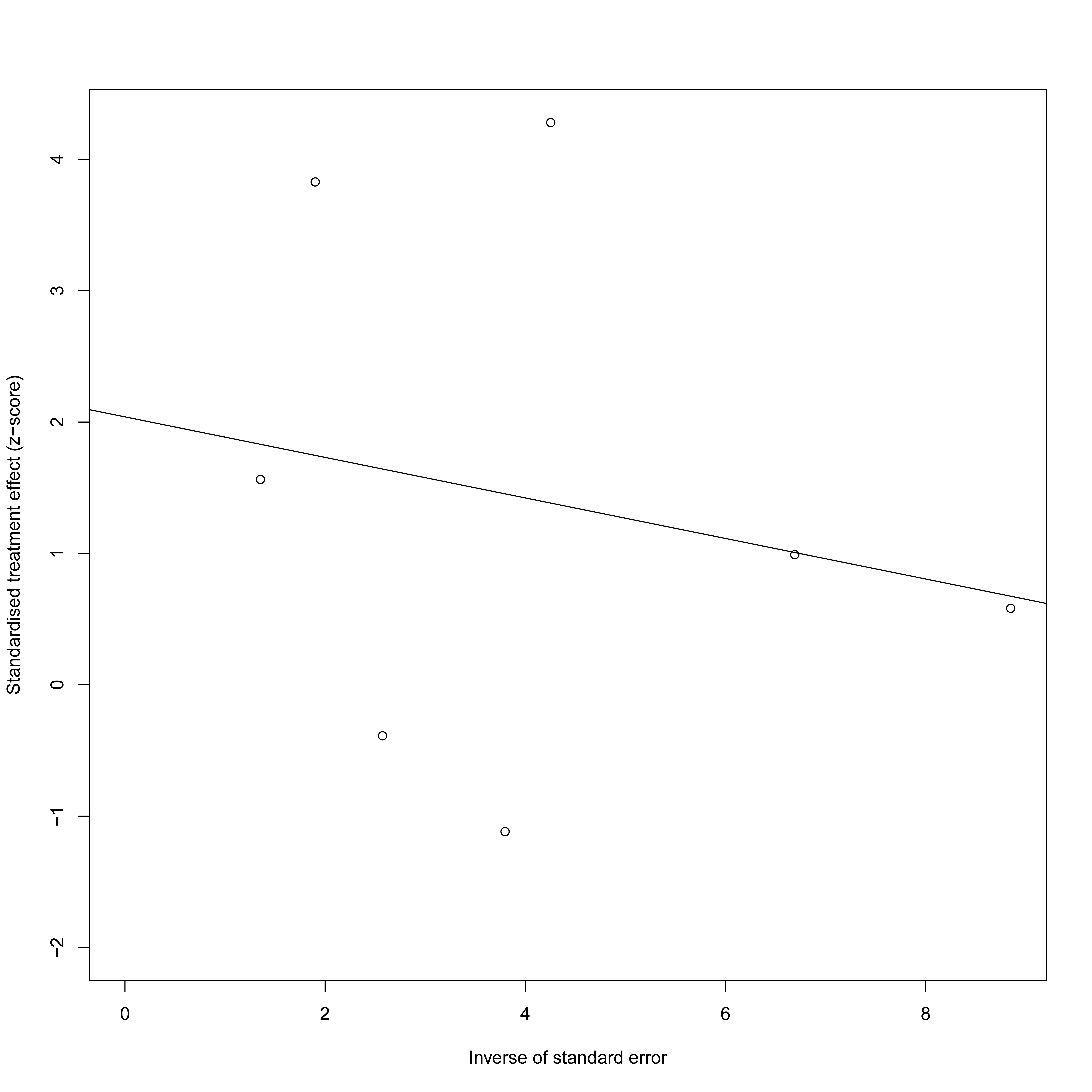 |
| 1. 90-day modified Rankin scale score of 0-1 (*P*=0.3479). |
| 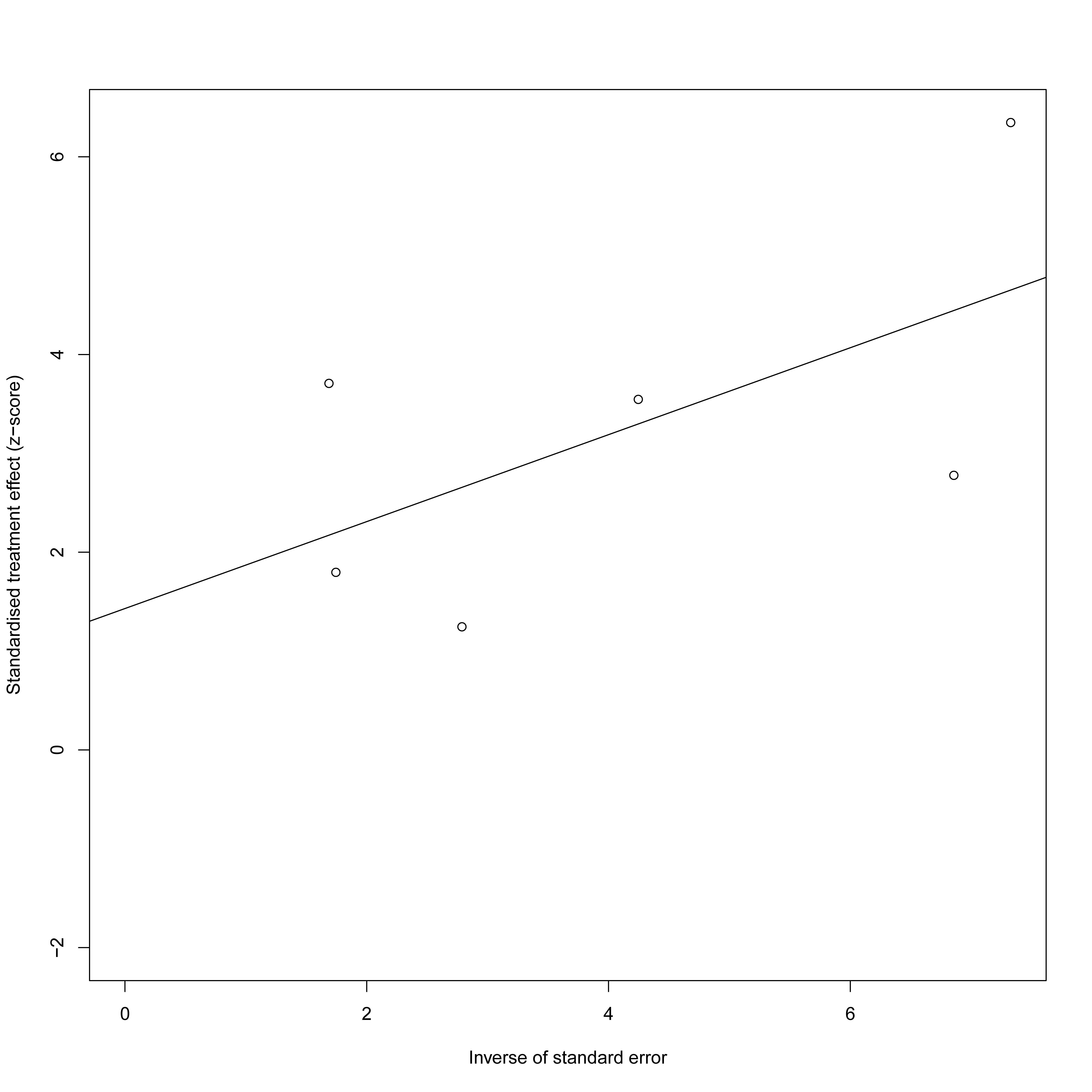 |
| 1. Symptomatic intracranial hemorrhage (*P*=0.2963). |
| 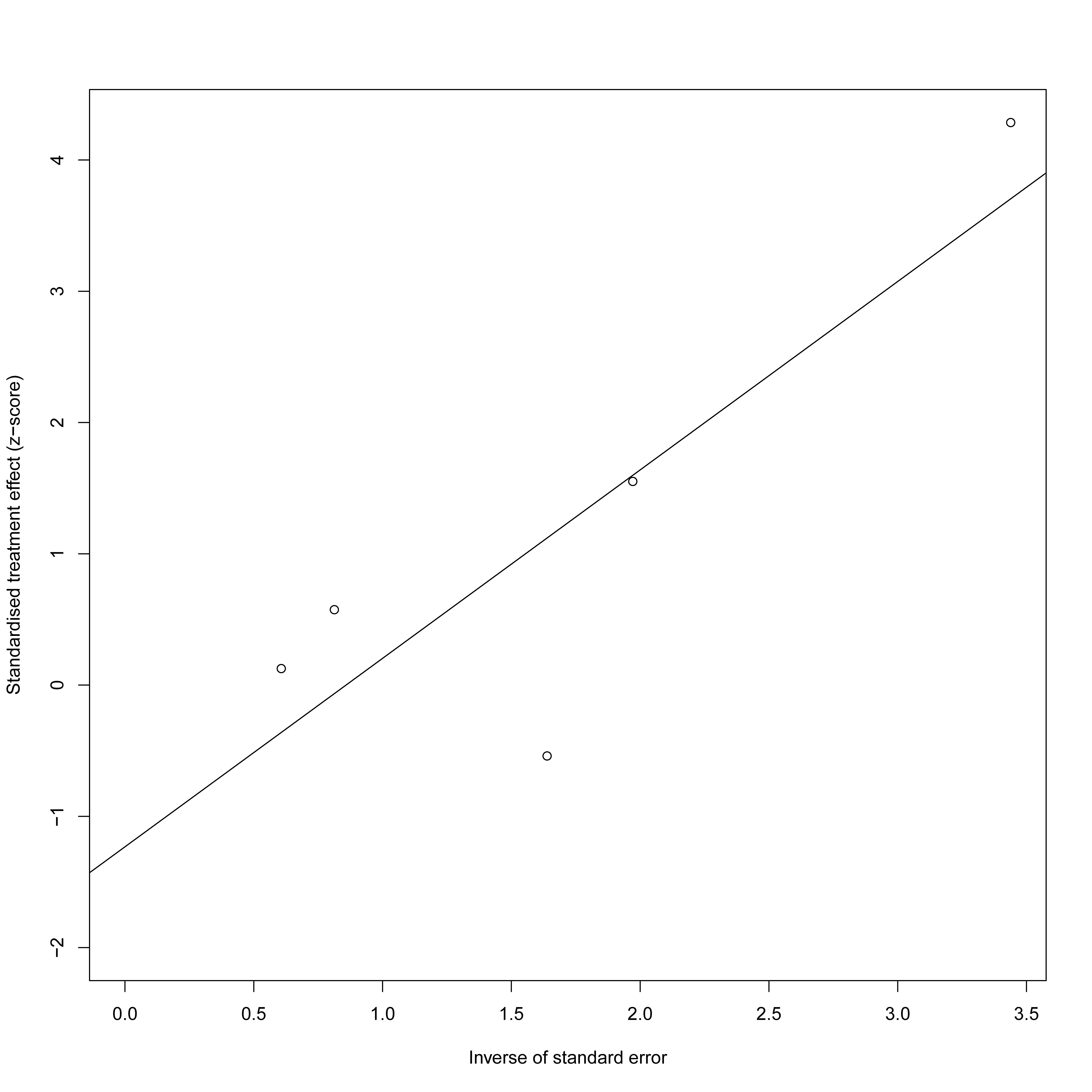 |
| 1. 90-day all cause mortality (*P*=0.5563). |
| 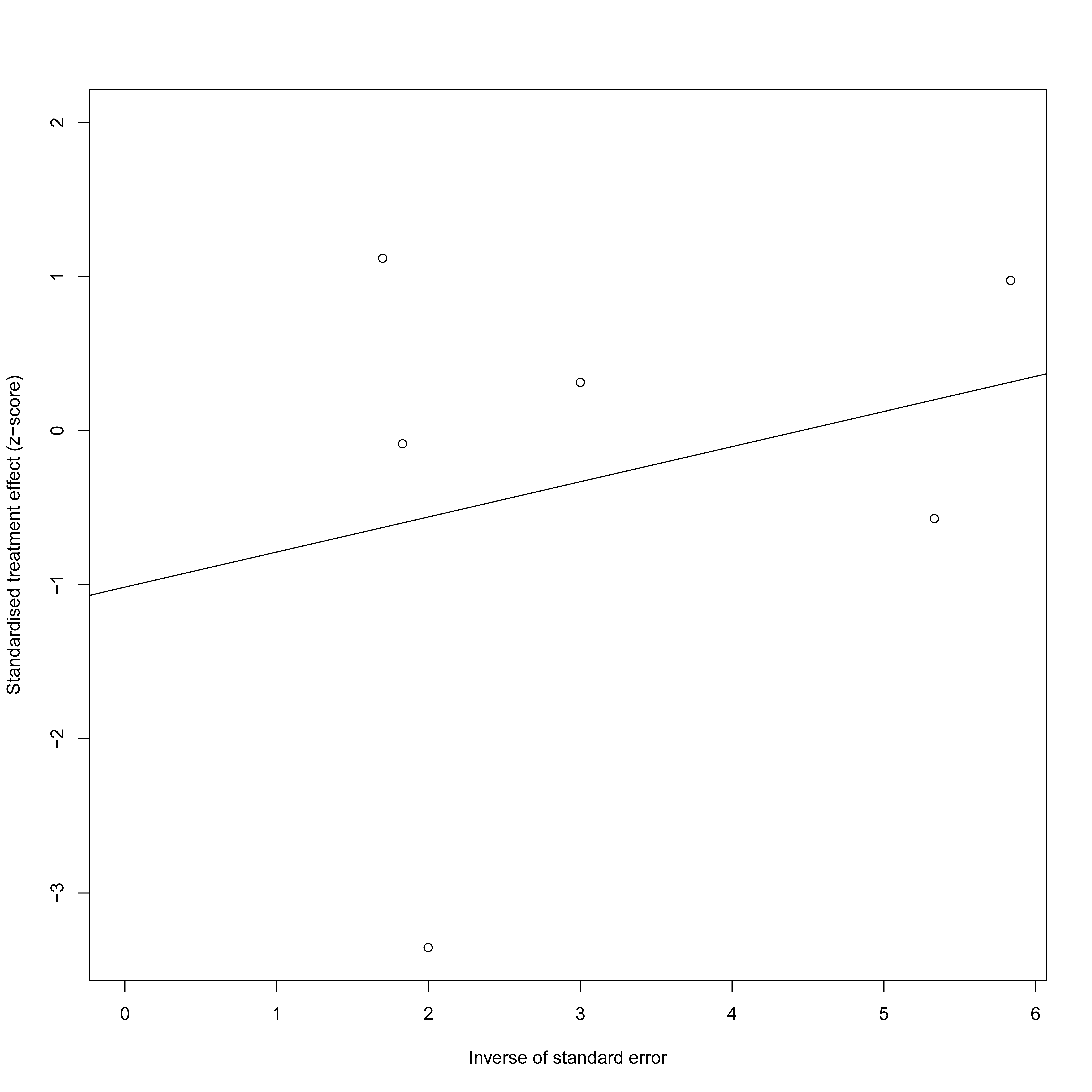 |
